# Effects of breastfeeding on body composition and maturational tempo in the rat

**DOI:** 10.1186/1741-7015-11-114

**Published:** 2013-04-29

**Authors:** Yonatan Crispel, Oren Katz, Dafna Ben-Yosef, Ze'ev Hochberg

**Affiliations:** 1 Division of Pediatric Endocrinology, Meyer Children's Hospital, Rambam Health Care Campus, Haaliya Street, Haifa 31096, Israel; 2 Endocrine Laboratory, Rambam Health Care Campus, Haaliya Street, Haifa 31096, Israel; 3 Rappaport Family Faculty of Medicine, Technion - Israel Institute of Technology, Efron Street, Haifa 31096, Israel

**Keywords:** growth, puberty, maturation, lactation, body composition, rat

## Abstract

**Background:**

Features of life history are subject to environmental regulation in the service of reproductive fitness goals. We have previously shown that the infant-to-childhood transition reflects the adaptive adjustment of an individual's size to the prevailing and anticipated environment.

**Methods:**

To evaluate effects of weaning age on life-history traits in rats, we repeatedly measured length and body mass index (BMI), as well as physiological development and sexual maturation in pups weaned early (d16), normally (d21) or late (d26). Males were bred to females of the same weaning age group for four generations.

**Results:**

Here, we show that the age at weaning from lactation regulates a rat's life history, growth, body composition and maturational tempo. We show that early-weaned rats developed faster than normal- or late-weaned rats; they are leaner and longer than late-weaned ones who are heavier and shorter. Early-weaned progeny develop more rapidly (that is, fur budding, pinnae detachment, eye opening); females show earlier vaginal opening and estrous and males show earlier onset of testicular growth. In generations 3 and 4, early-weaned rats bear larger litter sizes and heavier newborn pups. The entire traits complex is transmitted to subsequent generations from the paternal side.

**Conclusions:**

The findings presented here lend support to the proposition that the duration of infancy, as indexed by weaning age, predicts and perhaps programs growth, body composition, and the tempo of physiological development and maturation, as well as litter size and parity and, thereby, reproductive strategy.

## Background

A multiplicity of factors and forces, including genetic, environmental and stochastic ones, influence how organisms grow and develop. This is so with respect to reproductively significant traits, a primary focus of this report, such as maturational tempo and pubertal timing [1]. Life-history theory is a powerful evolutionary framework [2,3] for understanding a second focus of this essay: adaptive development plasticity, particularly with regard to growth- and metabolic-related strategies for transitioning from one life-history phase to the next [4-6].

When it comes to the environmental regulation of life history, the time of maximal developmental plasticity appears to be during the prenatal and early postnatal periods [6]. Indeed, the infancy-to-childhood transition (ICT) is often a time of heightened nutritional stress and mortality in humans, thereby representing a bottleneck for evolutionary forces. In social mammals, including apes, as well as in traditional human societies, the ICT is the time of weaning from breastfeeding, a third important focus here, demarcating the transition from maternal provision, protection and support to greater independence. Weaning from lactation is itself responsive to sex, stress and other environmental cues [7] that are presumed to inform the developing organism about risks and opportunities in its current and future environment. As such, the timing of the ICT has been hypothesized to reflect the adaptive adjustment of a species or an individual's size to the prevailing and anticipated environment, as the ICT is a major determinant of final adult height [5].

This view, that weaning is regulated by contextual conditions and serves to regulate life history in the service of fitness goals, led us to test experimentally in rats the proposition that the age of weaning, our index of ICT, provides cues for growth, maturation, developmental tempo and litter size, thereby affecting these outcomes. We also evaluate the proposition that such effects of weaning age are enhanced across generations in rats.

To evaluate effects of weaning age on life-history traits in rats, as well as on their offspring's development, we repeatedly measured length and BMI, as well as physiological development and sexual maturation in pups weaned early (d16), normally (d21) or late (d26). Due to concerns that the effects of maternal care on behavior could be reversed by cross-fostering [8,9], steps were taken to eliminate this possibility: weaning was accomplished through cross-fostering by non-lactating mothers, and separation was carried out on d30 for all. By the nature of that design, timing of weaning is confounded by time spent with a foster mother. Upon removal of lactating mothers, food was supplied *ad lib *as both chow and powder to ascertain its reach by all pups.

## Methods

### Animals

Gestating outbred Sprague-Dawley mother rats from timed pregnant colonies were housed at the Animal Facility of the Technion Faculty of Medicine (F0 generation). Delivered F1 generation offspring pups were diluted on Day 3 (d3) to include four female and four male pups for each mother. F1 males were bred to F1 females of the same weaning age group but from different litters, generating the F2 generation; F2 males were bred to F2 females to generate the F3 generation and the F3 were bred in the same manner to generate the F4 generations. No inbreeding or sibling crosses were generated. All animals were grown uninterrupted other than for weekly measurements until weaning on the designated d16, d21 or d26. The entire experiment from F0 to F2 was performed twice. On weaning day, mothers were removed and mothers that had weaned litters successfully in the recent month were introduced as foster mothers [10]. Upon weaning, both chow cubes and chow powder were introduced to cages to ascertain *ad lib *feeding by young pups. The number of female pregnancies used for replicates in each weaning group on each generation was three, to provide 12 males and 12 females per group

### Developmental milestones

Developmental milestones for infantile (pre-weaned) rats were observed daily from d7 to 20 [11]: incisor eruption (d7 milestone), fur budding (d11 milestone), eye opening (d13) and pinnae detachment (d15). Rat BMI was calculated as body weight over cubic rump-tail length (gr/cm2).

### Pubertal maturation

Vaginal opening was determined by supine observation as of d30, showing closed vaginal cavity before and opened vaginal cavity after. First estrus was determined as of d36 by observing a vaginal smear daily for 15 days between 9 and 10 AM. Vaginal smears were prepared by introducing a drop of distilled water into the vagina, collecting back and placing it on a clean slide after adding a drop of glycerin. Estrus phase was confirmed when the smear showed more than 50% cornified epithelial cells. Testicular size was determined daily as of d30 using a self-built orchidometer based on the human Prader orchidometer [12] with mock-ups ranging from 0.5 to 5 ml. Testes measuring more than 1 ml were considered pubertal.

### Body composition

For assessment of body composition in newborn rats, we used the Minispec live mice TD-NMR Analyzer (Bruker LF50, Ettlingen, Germany) - a mice magnetic resonance apparatus for animals up to 15 g [13]. The onboard electronics calculate whole-body water, fat and lean mass. Because of the design characteristics of the instrument, lean mass is most highly correlated with skeletal muscle. Unanaesthetized animals were placed into a cylindrical holder and inserted into a receiving port on the machine for less than two minutes, allowing scans in triplicate.

### Enteral glucose tolerance test [14]

The test was performed after six hours fasting around 2 pm. 0.1 g/ml glucose was given intragastric through a feeding tube to provide 1 g glucose for each 100 g body weight. A drop of blood was taken from the cut tail vein before the glucose load and after 15, 30, 45 and 60 minutes for the determination of blood glucose with a glucometer.

### Insulin Tolerance Test [14]

The test was performed on random-fed rats around 2 pm. The rats were injected with insulin (0.75 U/kg) in 0.1 ml 0.9% NaCl intraperitoneally. A drop of blood was taken from the cut tail vein before the injection of insulin and after 15, 30, 45 and 60 minutes for the determination of blood glucose with a glucometer.

### Rat GH and IGF1

Serum levels of rat GH and rat IGF1 were measured using the MG100 Rat/Mouse Growth Hormone and Rat IGF-I Quantikine ELISA Kit (R&D Systems, Biotest, Solihull, West Midlands, UK).

### Statistical analysis

Data were analyzed using an SAS program (SAS Institute Inc., Cary, NC. USA). The values were expressed as means and standard deviation (SD) or standard error of mean (SEM), as indicated. Statistical analysis was performed and the difference among the means of three groups was determined using two-way Analysis of Variance (ANOVA). A statistically significant difference was confirmed at *P *<0.05. We did not control for litter effects.

### Ethical approvals

All animal procedures have been approved by the Technion Animal Use and Care Committee and were performed under the supervision of an experimental animal veterinarian surgeon.

## Results

### Growth of early- or late-weaned animals

**Figure 1:**
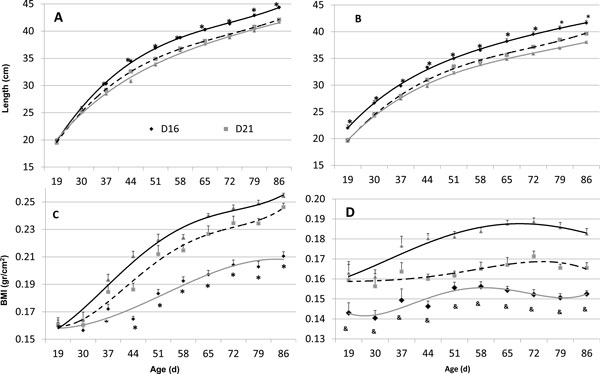
**Growth phenotype according to weaning age**. Length and body mass index (BMI) of rats of F1 generation according to weaning age day (d) 16, 21 or 26: Males (**A, C***) *and females (**B, D**) were measured for crown-tale length (A, B) and weight to give the BMI (g/cm2; C, D) from age d19 to 90. Statistically significant differences between d16 and d26 rats are indicated by (&) for *P *<0.02, (*) for *P *<0.001 with a two-way t-test. Mean ± SEM, *n *= 12 per group.

Results revealed that weaning age influences the length and adiposity of rats from infancy through adulthood, with short lactation resulting in a thin/long phenotype and long lactation in a short/heavy phenotype. Specifically, early-weaned (d16) F1 male and female rats were longer and late-weaned (d26) shorter relative to d21-weaned control animals. This was true from d30 to 90, with the difference emerging between d19 and 30 (Figure 1a, b). Long d16 rats were thinner by BMI and short d26 animals were heavier than d21-weaned control animals (Figure 1c, d). Food consumption varied between the groups, as did endocrine measurements. Average (± SD) adult daily food intake was 10.0 ± 0.3 g for d16 rats, 10.6 ± 0.2 g for d21 and 11.2 ± 0.3 g for the short/heavy d26 male rats (*P *<0.01 *vs*. d16). Although serum growth hormone (GH) of the two groups did not differ, serum IGF-1 did, being greater in late-weaned (short/heavy) rats (0.57 ± 0.20) as compared to early-weaned ones (0.38 ± 0.17 ng/ml, *P *= 0.028).

**Figure 2:**
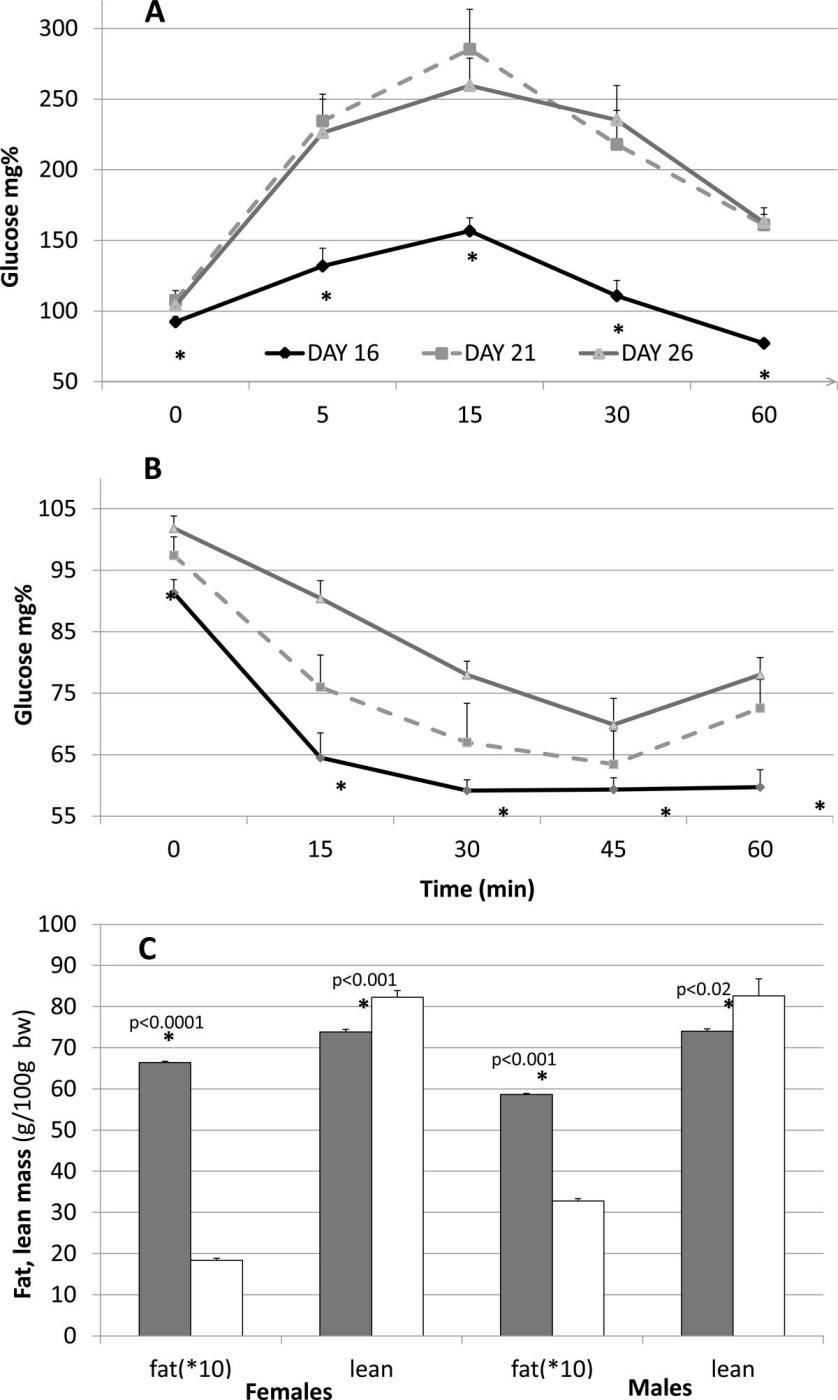
**Glucose tolerance, insulin sensitivity and pups body composition**. Glucose tolerance, insulin sensitivity and pups body composition according to weaning age d16, d21 or d26 in male adult F1 rats on d80 to 90. (**A**). Enteral glucose tolerance test: after six hours fasting, 0.1 g/ml glucose was given intragastic through a feeding tube to provide 1 g glucose for each 100 g body weight. (**B**) Insulin tolerance test: Rats were injected intraperitoneally with insulin (0.75 U/kg) and blood glucose was measured by glucometer. (**C**) For assessment of body composition, d10 rats of F2 generation, whose parents weaned on d16 (*dark bars*) or d26 (*white bars*), were tested by live-mice TD-NMR Analyzer. The onboard electronics calculated fat and lean (mostly muscle) mass per 100 g body weight (% fat is multiplied by 10 for clarity). Statistically significant differences between d16 and d26 rats are indicated by (*) for *P *<0.05 with a two-way t-test. Mean ± SEM, *n *= 6 per group.

In all growth parameters, the d16 group differed more than the d26 group from the d21 controls; therefore, we highlight especially the unique phenotype of early-weaned animals. The long/thin habitus of the d16-weaned group was associated in adulthood (d80 to 90) with greater glucose tolerance and insulin sensitivity, as compared to d26 rats, as determined by an enteral glucose tolerance test and an insulin tolerance test; Figure 2a, b shows these differences in males, with similar results emerging in females

The long/thin phenotype of early-weaned F1 animals was sustained across generations but not intensified, with the same being true of the short/heavy phenotype of late-weaned F1 animals. This was determined after F1 and F2 male rats were mated on d60 to 70 with F1 to F2 females of the same weaning age group from different litters, with the weaning age of each group maintained for F2 to F3 pups.

### Infantile body composition

To test the hypothesis that being a member of the late-weaned lineage (short/heavy/insulin resistant) is associated with infantile wasting, body composition of F3 pups was assessed on d10 by TD-NMR live mice Analyzers NMR (Minispec, Bruker LF50, Ettlingen, Germany), where lean mass is highly correlated with skeletal muscle [13]. Offspring of early-weaned d16 parents, compared to d26 offspring had greater d10-body-fat mass (*P *<0.0001 and 0.001 for females and males, respectively) and smaller lean body mass (*P *<0.001 and 0.05, respectively; Figure 2c).

### Developmental tempo

**Figure 3:**
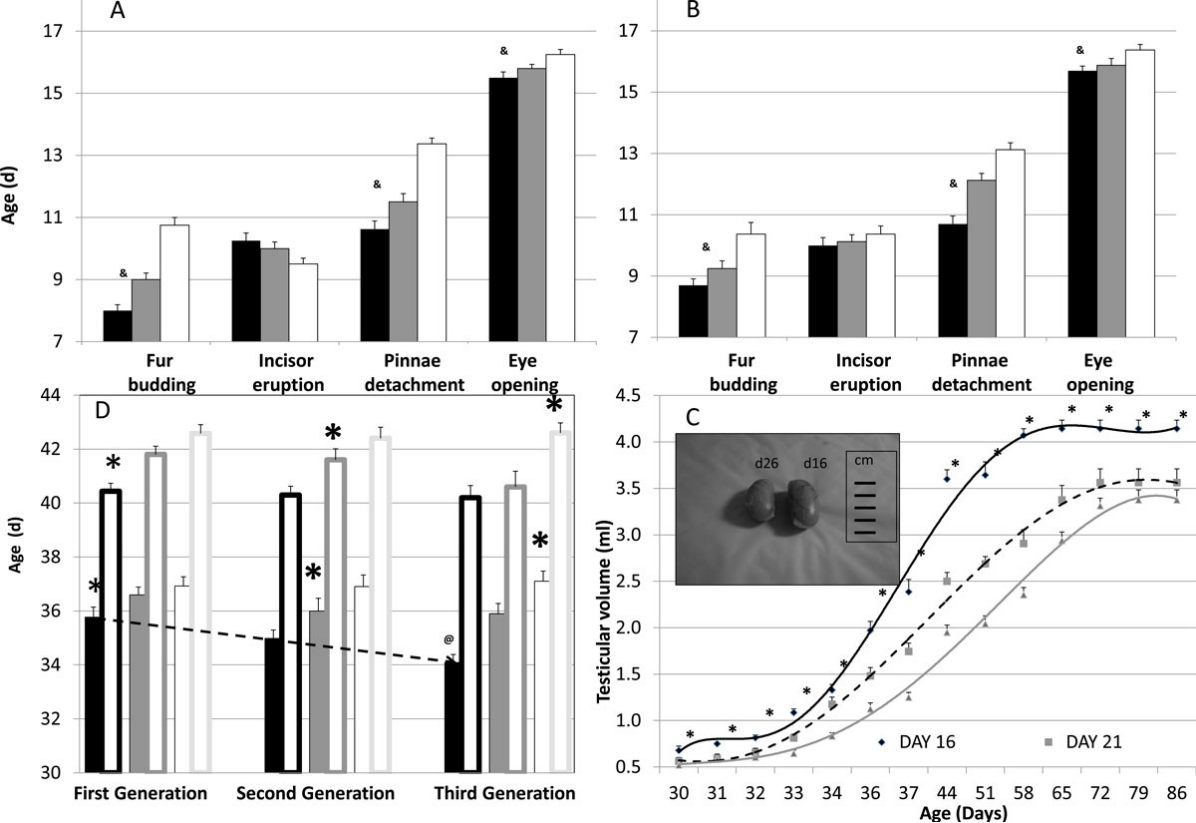
**Infantile development and sexual maturation**. Infantile development and sexual maturation according to weaning age in male (A, C) and female rats (B, D). (A, B) Developmental milestone for infantile (pre-weaned) rats were observed daily from d7 to 20: fur budding (d9 milestone), incisor eruption (d10), pinnae detachment (d12) and eye opening (d16). (C) Testicular size was determine daily as of d30 using a self-built orchidometer based on the human Prader orchidometer with mock-ups ranging from 0.5 to 5 ml. Gonadarche was defined when testicular volume reached 1 ml. (D) The initial day of vaginal opening (*solid bars*) was determined as of d30. First estrous (*open bars heavy frames*) were determined as of d36 by observing vaginal smears for more than 50% cornified epithelial cells. Statistically significant differences between d16 and d26 rats are indicated by (&) for *P *<0.05, (*) for *P *<0.001 with a two-way t-test. Dotted line shows enhancement of the traits from F1 to F3; (@) for *P *<0.01. Mean ± SEM, *n *= 8 to 12 per group.

Research on rodents shows that an animal's early rearing environment regulates pubertal, sexual and reproductive development [15]. To test the hypothesis that weaning age programs the tempo of development, F1 to F3 animals were observed from d7 to 20 for the following infantile developmental milestones [11]: fur budding (mean d9.2 milestone for control group d21), incisors eruption (d10.2), pinnae detachment (d12.1) and eye opening (d15.9). Whereas F1 animals showed no difference across the original age-of-weaning groups (data not shown), F2 offspring of early-weaned parents accelerated their pre-weaning infantile development as compared to offspring of late-weaned parents, showing earlier fur budding, pinnae detachment and eye opening (all *P *<.05), but not incisor eruption; Figure 3a, b). This difference in developmental tempo was sustained for F3 to F4 pups.

### Reproductive strategy

The age at sexual maturation plays a central role in reproductive strategy. We thus predicted and found that early-weaned F2 females showed earlier vaginal opening (*P *<0.01) and estrous (*P *<0.01) and early-weaned males showed earlier onset of testicular growth (*P *<0.05) and attainment of maximal testicular volume (*P *<0.05) compared to late-weaned rats (Figure 3c, d). Moreover, adult d16 animals had larger testes compared to the other two groups (*P *<0.001). These traits were enhanced from F2 to F4 generations in both female (Figure 3c) and male rats (data not shown).

**Figure 4:**
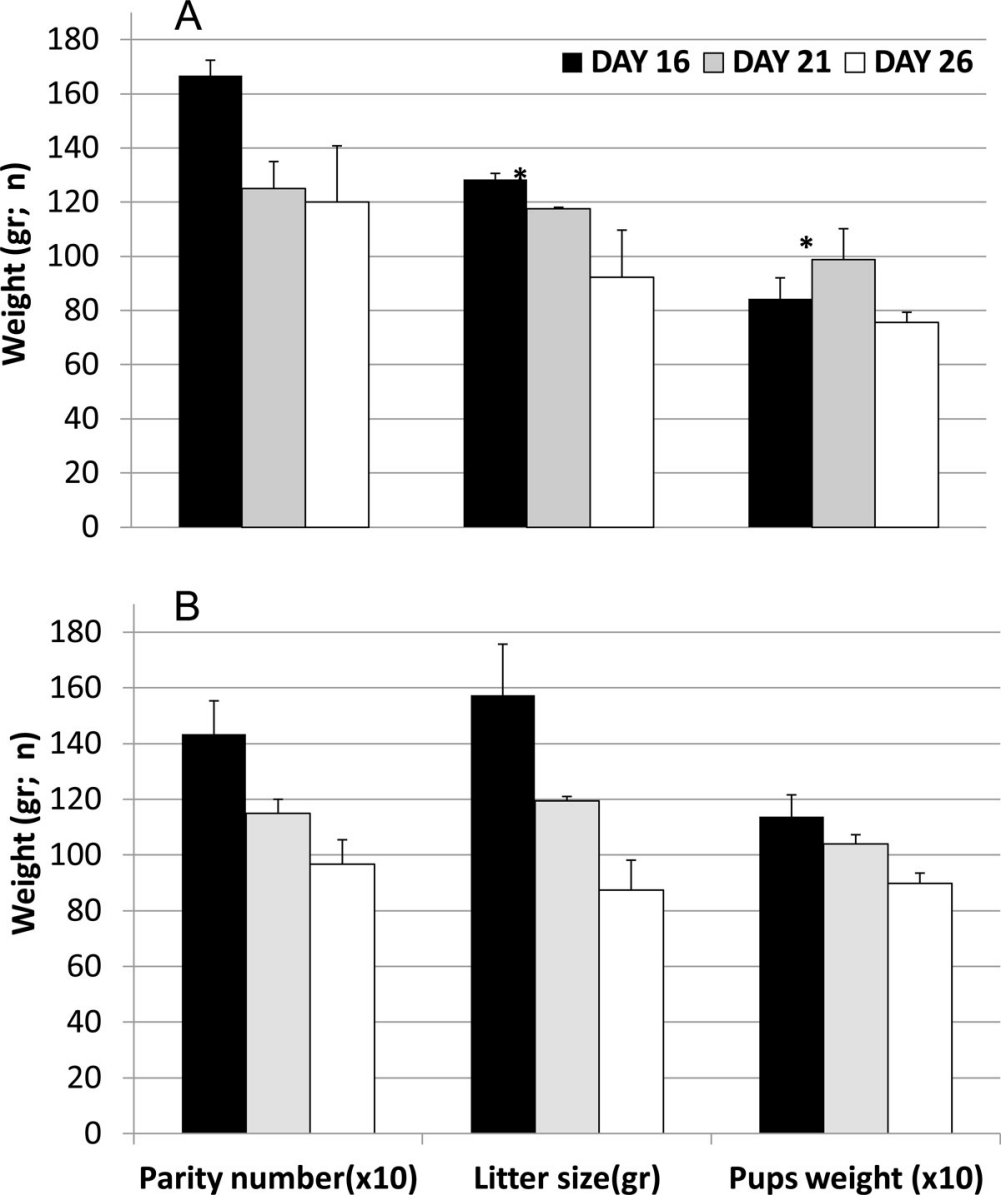
**Parity number, litter size and d1-calculated weight**. Parity number, litter size and d1-calculated weight according to weaning age in F3 (A) and F4 generations (B). Statistically significant differences between d16 and d26 rats are indicated by (*) for *P *<0.05 with a two-way t-test, and (&) for *P *<0.03. Mean ± SEM, *n *= 3 mothers per group.

To further test for effects on reproductive-strategy-related outcomes, we studied parity numbers and d1 litter size of early- and late-weaned parents (F1) and grandparents (F2), predicting that early-weaned progeny would themselves bear more offspring than the progeny of later-weaned F1 rats. In the primiparous F3 and F4 rats, parity number of early-weaned was greater than late-weaned animals (*P *<0.05 and <0.01, respectively) (Figure 4a, b). The d1 litter size was greater in early-weaned than late-weaned in F3 (*P *<0.01) and F4 (*P *<0.01). Finally, we discovered that d16 were heavier than d26 pups in F3, *P *<0.02, and in F4 litter, *P *<0.04 upon examining the mean weight of pups for each group after dividing d1 litter size by parity number.

### Phenotypic discordance upon paternal or maternal transmission

**Figure 5:**
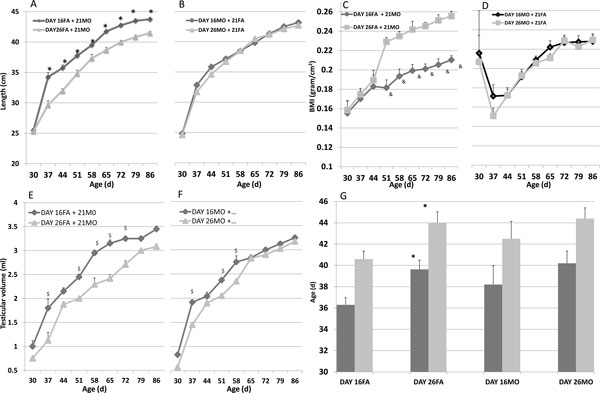
**Phenotypic discordance upon paternal or maternal transmission**. Rats of the F1 generation were mated to cross fathers weaned on d16 (*dark lines*) or d26 with mothers weaned on d21 (**A, C, E**), and mothers weaned on d16 *(dark lines*) or d26 with fathers weaned on d21 (**B, D, F**): F2 Offspring were measured for crown-tale length (*cm; *A, B), weight to give the BMI (g/cm2; C, D), testicular volume (ml; E, F) and vaginal opening (day; *solid bars*) and first estrous (day; *gray bars*) (G). Statistically significant differences between d16 and d26 rats are indicated by ($) for *P *<0.04, (*) for *P *<0.01, (&) for *P *<0.001 with a two-way t-test. Mean ± SEM, *n *= 12 per group.

When we evaluated the relative influence of mothers' and fathers' own age of weaning (as pups) on the weaning-related phenotype of their offspring - after mating (a) F1 females that had weaned on d21 with F1 males that had weaned on d16 or d26 and (b) F1 males that had weaned on d21 with F1 females that had weaned on d16 or d26 - only paternal influence proved evident (Figure 5): growth was faster (*P *<0.01) and BMI was smaller (*P *<0.001) in F2 offspring of F1 fathers weaned on d16 as compared to fathers weaned on d26 (Figure 5A-D); and testicular growth was greater (*P *<0.001) and vaginal opening (*P *<0.01) and estrous were earlier (*P *<0.01) in F2 offspring of F1 fathers, weaned on d16 as compared to fathers weaned on d26 (Figure 5E-G).

## Discussion

The findings presented here on rats lend support to the proposition that the duration of infancy, as indexed by weaning age, predicts and perhaps programs growth, body composition, and the tempo of physiological development and maturation, as well as litter size and parity, and, thereby, reproductive strategy.

Two important environmental cues for development of the young animal (and humans) are 1) the care-giving behaviors of their parents, which can be used as a predictive indicator of the security of their environment, and 2) provision of nutrition during the immediate postnatal period, which may predict nutritional availability during future life. The resultant patterns will be transmitted trans-generationally [16,17]. In view of prior evidence that weaning in rats has unique and critical effects on adult behavior [18], the data presented here indicate that weaning age influenced development in a manner consistent with an insecure, fast, life-history strategy (15), including accelerated growth, development and maturation, long/thin stature and, in subsequent generations, large litter size. In contrast, prolonged lactation influenced development in a manner consistent with a secure, slow, life-history strategy, including slower growth, development and maturation, short/overweight stature, and, in subsequent generations, small litter size. Yet, under natural conditions, early weaning might well be associated with more robust nutritional conditions that lead to more rapid pup growth and an earlier attainment of an appropriate size for independence. Similarly, in humans living in subsistence ecological contexts, early weaning is associated with better nutritional conditions.

The growth-promoting effect in rats of short infancy is much in line with human observations on the growth impact of early ICT [4,5]. We have proposed that adult size is determined to an important extent during transition from infancy to childhood. This transition is marked by a growth spurt. A delayed transition has a lifelong impact on stature and is responsible for 44% of children with short stature in developed countries, and many more in developing countries. This conformed with the theory of an evolutionary adaptive strategy of plasticity in the timing of transition from infancy into childhood in order to match the prevailing energy supply: humans evolved to withstand energy crises by decreasing their body size, and evolutionary short-term adaptations to energy crises trigger a predictive adaptive response that modify the transition into childhood, culminating in short stature [1,6].

The apparent tempo-accelerating effect in infantile developmental milestones of parental short lactation in the rat indicates a developmental signal transmitted across generations and seemingly aimed to prepare the young animal for independence for provision and protection upon weaning. This raises the interesting question of exactly how short lactation accelerates development, as we have provided evidence that it does, not of the underlying neurobiological mechanisms involved. Future work will need to illuminate such processes. In any event, it is of interest that early sexual maturation and large litters in animals are in line with an accelerated life history tempo brought about by shortening of the infancy stage. In swine, sows' parity number and litter size increase as lactation is prolonged from 8 to 13 to 18 to 21 days, but decrease if lactation is further prolonged to 22 to 25 days [19], in agreement with the rat findings.

Asymmetries in the costs and benefits of parental investment for mothers and fathers result in family conflict over their offspring's growth [20]. In species where females provide most resources before and after birth, the resolution of this conflict may be influenced by genes expressed in mothers and by maternally and paternally inherited genes expressed in offspring [21]. Here we show that the weaning-related trait is transmitted from the paternal side; offspring of fathers but not of mothers who weaned early were longer, thinner and had earlier sexual maturation. Previous work in mice showed that differences in litter size are determined by paternal genotype, whereas differences in provisioning are under maternal control, suggesting that there is antagonistic coadaptation of maternal and paternal effects on distinct life-history traits [21]. These results are consistent with a negative correlation that we discerned between the age of the infancy-childhood growth transition and fathers' height but not mothers' heights [6], leading us to conclude that the trans-generational transmission of transition age appears paternally derived.

The fact that the glucose intolerance and insulin resistance were related to delayed weaning is considered a response to being overweight. The thrifty phenotype theory suggests that intrauterine wasting and early infantile growth acceleration are associated with later acquisition of obesity and insulin resistance [22]. Indeed, on d10 offspring of late weaned animals, which are to become overweight as adults, had diminished adipose tissue, suggesting that the adult phenotype may be influenced by infantile expansion of the adipose tissue [23].

Considered together, the animal results reported support a conditional-adaptational view of individual differences in the infantile stage: developmental tempo and pubertal maturation are accelerated adaptively in response to shortening of infancy to allow for juvenile independence upon early weaning (development) and earlier reproduction (sexual maturation) [24].

## Conclusions

In the discussion of nature and nurture, our results lend support to a major impact of a conditional adaptation within a single generation, and provide insight into the role of plasticity over one-to-three generations in reproductively important traits such as size, developmental and maturational tempo. The notion that developmental tempo is regulated by weaning age to produce such contrasting phenotypes, and that fathers transmit the trait, has significant implications for evolutionary biology as well as to child growth and maturation in a changing society.

## Abbreviations

BMI: Body mass index; ICT: the infancy-to-childhood transition.

## Competing interests

The authors declare that they have no competing interests.

## Authors' contributions

YC and ZH designed and YC, OK, DBY and ZH performed the study. YC and ZH contributed to writing of the manuscript. All authors have read and approved the manuscript for publication.

## Pre-publication history

The pre-publication history for this paper can be accessed here:

http://www.biomedcentral.com/1741-7015/11/114/prepub
